# Canaloplasty in Open-Angle Glaucoma Surgery: A Four-Year Follow-Up

**DOI:** 10.1155/2014/469609

**Published:** 2014-01-16

**Authors:** Paolo Brusini

**Affiliations:** Department of Ophthalmology, Santa Maria della Misericordia Hospital, Piazzale S. Maria della Misericordia 15, 33100 Udine, Italy

## Abstract

Canaloplasty is a new nonperforating surgical technique for open-angle glaucoma, in which a microcatheter is inserted within Schlemm's canal for the entire 360 degrees. A 10-0 prolene suture, which is tied to the distal tip of the microcatheter, is then positioned and left tensioned in Schlemm's canal, thus facilitating aqueous outflow through natural pathways. A small amount of viscoelastic agent is delivered in Schlemm's canal while the catheter is withdrawn. The mid-term results are very promising. Based on our cohort of 214 patients, the percentages of eyes that obtained postoperative IOP ≤ 21 mmHg, ≤18 mmHg, and ≤16 mmHg with or without medical therapy after 2 and 3 years were 88.7%, 73.7%, and 46.2% (2 years); 86.2%, 58.6%, and 37.9% (3 years), respectively. The most frequent complications observed included hyphema; descemet membrane detachment; IOP spikes; and hypotony. The advantages of canaloplasty over trabeculectomy include (1) no subconjunctival bleb; (2) no need for antimetabolites; (3) fewer postoperative complications; and (4) a simplified follow-up. The disadvantages include the following: (1) a long and rather steep surgical learning curve; (2) the need of specific instruments; (3) average postoperative IOP levels tend not to be very low; and (4) impossibility to perform the entire procedure in some cases.

## 1. Introduction

**Figure 1:**
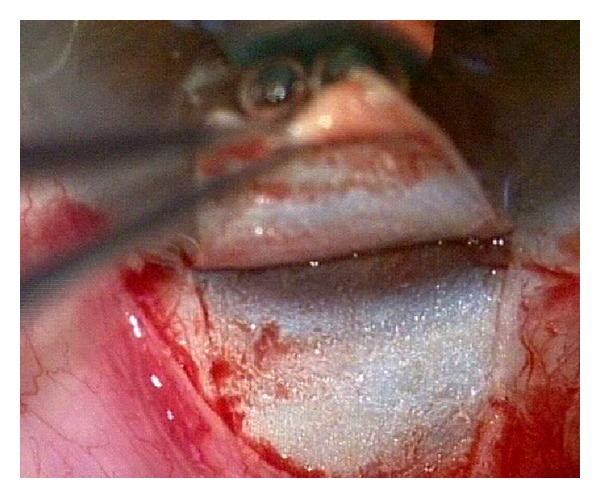
Dissection of the superficial sclera flap.

**Figure 2:**
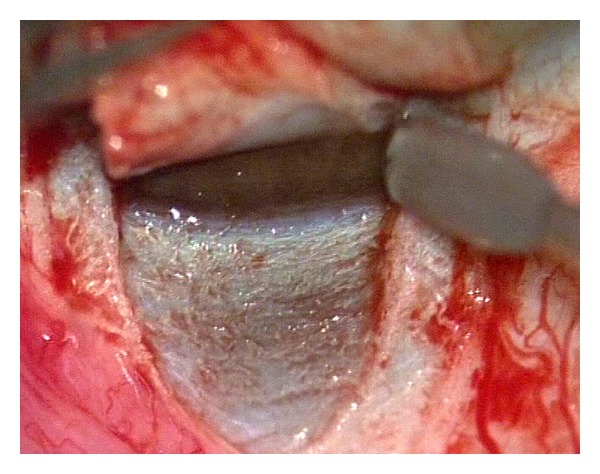
Deep scleral flap.

**Figure 3:**
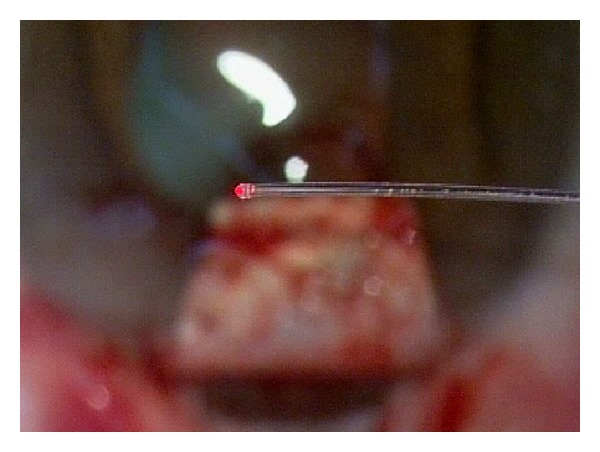
Microcatheter connected to a laser flickering red light source.

**Figure 4:**
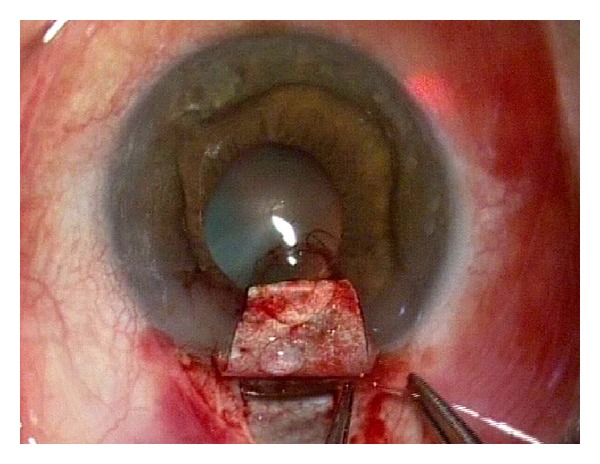
Cannulation of the Schlemm's canal. The distal tip can clearly be seen through the sclera (red point at the upper right corner).

**Figure 5:**
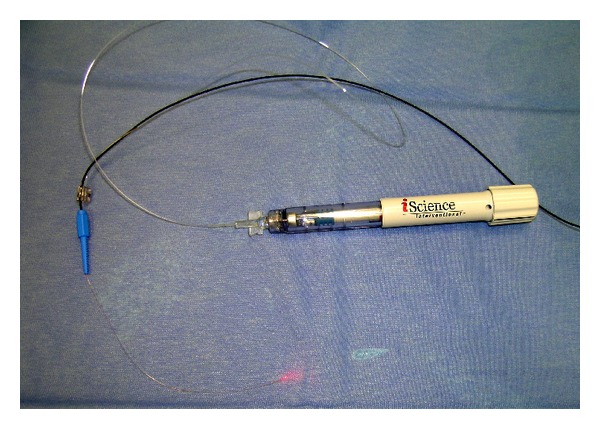
Screw-driven syringe connected to the microcatheter.

**Figure 6:**
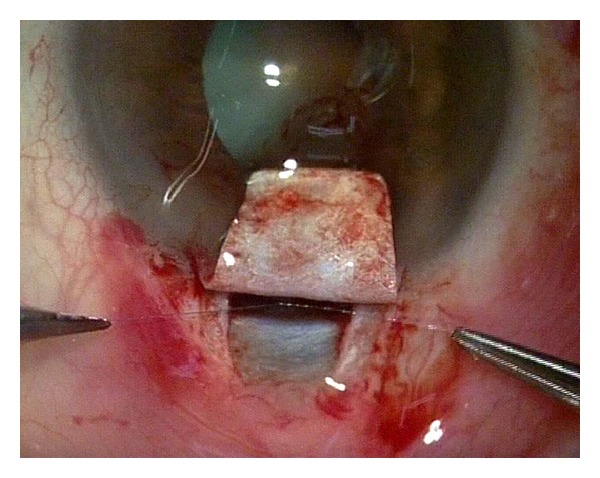
Prolene 10-0 suture.

**Figure 7:**
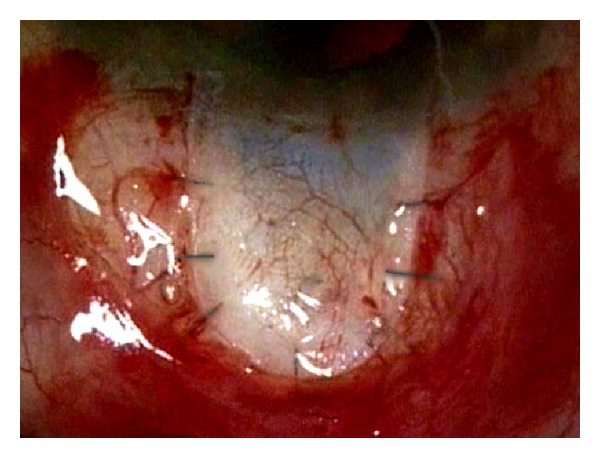
Watertight suture of the superficial sclera flap.

**Figure 8:**
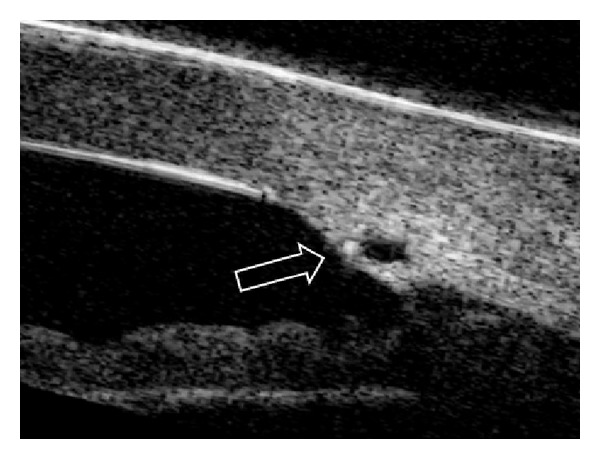
UBM image showing the enlarged Schlemm's canal and the prolene suture within the canal (arrow).

Canaloplasty is a relatively new nonperforating blebless technique, quite similar to Stegmann's viscocanalostomy [1], in which a 10-0 prolene suture is positioned and tensioned within Schlemm's canal after the injection of a small amount of high viscosity sodium hyaluronate, thus facilitating aqueous outflow through natural pathways (collectors channels and aqueous veins) [2–12]. Surgery starts with a fornix-based conjunctival flap and a 4 × 4 mm superficial scleral flap, similar to that performed in deep sclerectomy, which is dissected forward into clear cornea for 1.5 mm (Figure 1). A deep scleral flap is then sculpted (Figure 2), and Schlemm's canal is opened and deroofed by the removal of the inner wall, which is performed after paracentesis in order to lower the IOP, thus reducing the risk of perforation of the trabeculodescemet membrane. The deep scleral flap is removed and the two ostia of the canal are dilated with high molecular weight hyaluronic acid (Healon GV), similarly to a viscocanalostomy. A 200 micron microcatheter (iTrack by iScience Interventional, Menlo Park, CA, USA), which is connected to a laser flickering red light source for an easy identification of the distal tip through the sclera (Figure 3), is then inserted and pushed forward within Schlemm's canal for the entire 360 degrees (Figure 4) until it comes out of the other end of the of the canal opening. A single or double 10-0 prolene suture is then tied to the distal tip and the microcatheter is withdrawn and pulled back through the canal in the opposite direction. A small amount of viscoelastic agent is delivered in Schlemm's canal at every two or three clock hours while the catheter is withdrawn with the aid of a special screw-driven syringe (Figure 5). The suture is then knotted under tension in order to inwardly distend the trabecular meshwork (Figure 6). The superficial scleral flap is tightly sutured with 5 to 9 10-0 vicryl (or nylon) sutures to ensure a watertight closure preventing any bleb formation (Figure 7). The conjunctival flap is then sutured with 10-0 vicryl sutures. During the learning curve, high-resolution 80 mHz ultrasound biomicroscopy (*iUltrasound, iScience* Interventional, Menlo Park, CA, USA) may be useful for verifying that the suture is properly positioned and tensioned in the canal (Figure 8).

## 2. Material and Methods

The study was in compliance with the tenets of the Helsinki's Declaration, and informed consent was obtained from all participants prior to testing. The study was in compliance with Institutional Review Boards (IRBs) and HIPAA requirements of the Azienda Ospedaliero-Universitaria “S. Maria della Misericordia,” Udine, Italy. Glaucomatous patients were recruited from the Glaucoma Outpatient Centre of the Department of Ophthalmology of the S. Maria della Misericordia Hospital, Udine, Italy.

Our mid-term results on canaloplasty are based on 256 eyes from 224 patients affected by open-angle glaucoma under maximum tolerated medical therapy (189 primary open-angle glaucomas, 53 pseudoexfoliation glaucomas, 10 juvenile glaucomas, and 4 pigmentary glaucomas), with a mean age of 63.5 ± 14 years (ranging from 33 to 88 years) and a follow-up of up to 5 years (mean 20.3 months ±10.6). All but two cases (that underwent general anesthesia due to the preference of the patient) underwent canaloplasty under local anesthesia (peribulbar injection of carbocaine and lidocaine). All patients underwent postoperative local medical treatment with levofloxacin drops 4 times daily for 1 week and dexamethasone drops 4 times daily for 7 days followed by diclofenac drops four times daily for 1 month. The definition of “complete” success was based on three different criteria: postoperative IOP ≤ 21 mmHg, ≤18 mmHg, and ≤16 mmHg without any medical treatment. When the same IOP levels were obtained with medical treatment, the success was defined as “qualified.” The full procedure could not be performed in 42 eyes (16.4%), either due to a large perforation of trabeculodescemet membrane with iris prolapse (2 eyes) or to the impossibility of cannulating the full 360° of Schlemm's canal (40 eyes) due to anatomical obstacles and/or other intraoperative complications, such as the misdirection of the microcatheter in the anterior chamber. YAG laser goniopuncture was performed after 2 to 12 months in 26 eyes (12.1%), which were included in the analysis. In 14 eyes (6.5%), microperforation of the trabeculodescemet membrane occurred; however, Schlemm's canal cannulation and successful canaloplasty could still be performed; these eyes were thus included in the analysis. Seventeen patients (7.9%) later underwent trabeculectomy 3 to 58 months after canaloplasty due to poor IOP control and were thus considered as “unsuccessful.”

A 80 mHz UBM was used for measuring the suture tension 3 months after canaloplasty in 40 consecutive patients.

The Heidelberg Retina Tomograph (HRT) cornea module was used in all patients in order to study the episcleral vessels before and one month after surgery.

## 3. Results

**Figure 9:**
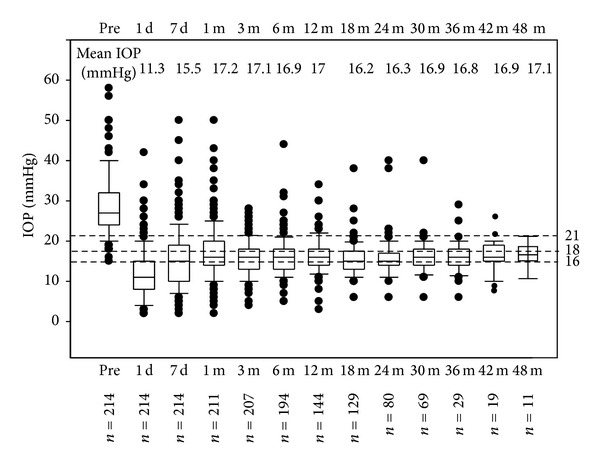
Box-plot representation of IOP values over 48 months of follow-up.

**Figure 10:**
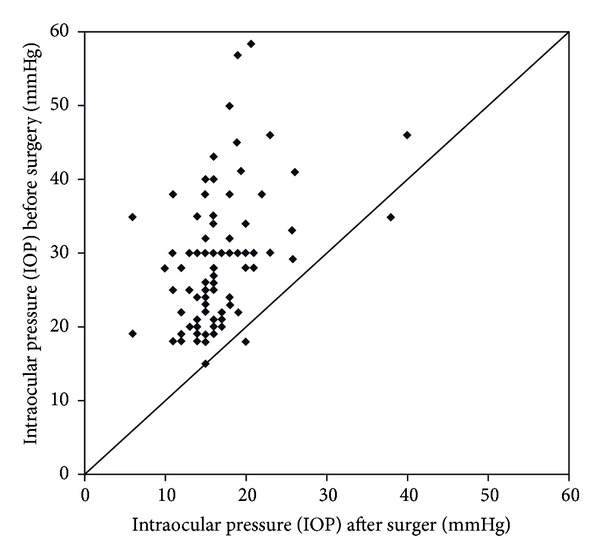
Scatter plot of IOP values before and after canaloplasty after 2 years (80 eyes).

**Figure 11:**
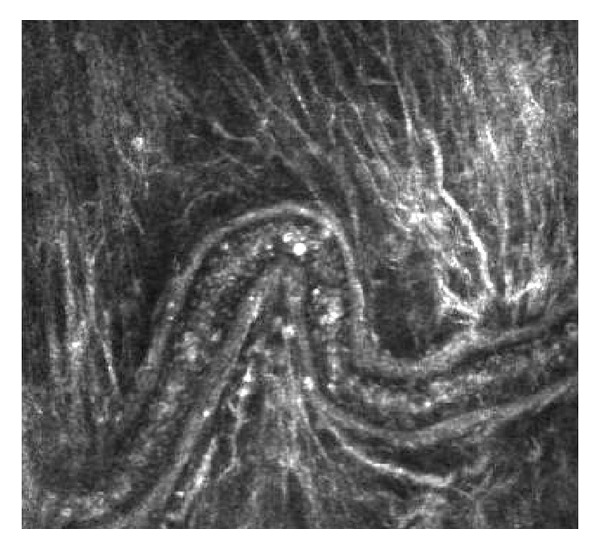
Enlarged aqueous vein after canaloplasty (Heidelberg Retina Tomograph cornea module).

**Figure 12:**
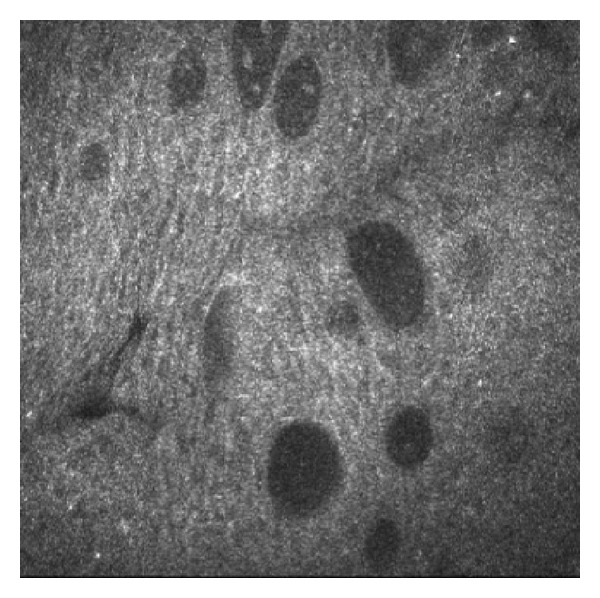
Conjunctival microcysts after canaloplasty.

The entire surgical procedure of canaloplasty was properly completed in a total of 214 eyes from 185 patients, which were considered in the statistical analysis. The preoperative mean IOP was 29.4 ± 7.9 mmHg (ranging from 18 to 60 mmHg). After excluding 17 eyes that later underwent trabeculectomy, the mean IOP at the last control was 17.0 ± 4.2 mmHg (ranging from 10 to 29 mmHg), with a mean IOP reduction of 42.2%. The results during the follow-up and the success rates after 1, 2, and 3 years are shown in Tables 1 and 2. The pre- and postoperative IOP values are shown with bar diagrams and scatter plots (Figures 9 and 10). The number of medications used before canaloplasty and at the 1-, 2-, and 3-year follow-ups was 3.3 ± 0.9, 0.7 ± 1.2, 1.1 ± 1.3, and 1.3 ± 1.5, respectively. The early postoperative complications are listed in Table 3. A transient decrease in visual acuity was commonly observed during the first two weeks after canaloplasty, due to an induced with-the-rule astigmatism. A late IOP rise was observed in 17 cases (7.9%). Postoperative UBM showed a good suture tension (>grade 1.5) in all cases, with exception to 2 eyes. The Heidelberg Retina Tomograph (HRT) cornea module clearly showed postoperative enlargement of aqueous veins and episcleral vessels in all successful eyes (Figure 11). Moreover, the same technology showed a significant increase in the density and surface of conjunctival microcysts (Figure 12).

## 4. Discussion

One of the most interesting and exciting characteristics of canaloplasty is that this procedure, unlike traditional trabeculectomy, works without the need of a filtering bleb, which is usually absent [13]. The vast majority of patients tend to have a perfectly normal looking eye after a few weeks, without any ocular discomfort. Although there is limited current literature that compares these two surgical techniques [14], canaloplasty should be proposed in patients with mild to moderate glaucoma, in which the target IOP is not too low. The procedure may prove to be unsuccessful in a small number of eyes, which is probably due to a nonreversible collapse of collector channels or other outflow pathways that cannot be enlarged due to anatomical factors.

The exact mechanisms behind canaloplasty is not perfectly known; however, the most likely explanation may be the permanent enlargement of Schlemm's canal and of the collectors channels. The increase of conjunctival microcysts after canaloplasty, which were evident after surgery with the HRT cornea module, could indicate enhanced aqueous humor filtration across the sclera and conjunctiva, thus representing adjunctive mechanisms for IOP decrease [15].

The best indications for canaloplasty include (1) primary open-angle glaucoma; (2) pseudoexfoliation glaucoma; and (3) pigmentary glaucoma. Canaloplasty can also be successfully performed in patients with failed trabeculectomy in which Schlemm's canal has been left undamaged from previous filtrating surgeries [16].

The contraindications for canaloplasty include (1) angle-closure glaucoma; (2) narrow-angle glaucoma (even if some eyes can be still considered after a laser or surgical iridectomy); (3) neovascular glaucoma; (4) posttraumatic glaucoma; (5) eyes with interruption or damage to Schlemm's canal due to previous ocular surgery or extensive laser trabeculoplasty with peripheral anterior synechiae; (6) ocular hypertension due to an increased episcleral venous pressure; and (7) other forms of secondary glaucomas.

The effect of canaloplasty on IOP appears to be correlated, at least in part, to the suture tension, as previously reported by other authors [2]. In our cases, a good suture tension was demonstrated in all cases but two, using a 80 mHz UBM. It is not known, however, whether or not there is a direct relationship between the tightness of the suture and postoperative outcomes.

The advantages of canaloplasty over trabeculectomy include the following: (1) subconjunctival bleb formation in not required; (2) antimetabolites are not needed; (3) faster visual rehabilitation after surgery; (4) fewer and simplified postoperative follow-ups; (5) limited postoperative complications; and (6) postoperative results and IOP levels tend to be stable over time (at least up to five years based on our current cohort).

The disadvantages includethe following; (1) long and rather steep learning curve; (2) need of specifically designed (and expensive) instruments; (3) average postoperative IOP levels tend not to be very low; and (4) impossibility to cannulate Schlemm's canal in about 10 to 15% of eyes (based on our results). In these cases, the procedure can easily be converted into either a deep sclerectomy or a viscocanalostomy, which tend to show good postoperative results (data not shown). It is important to note that the surgeon should avoid forcing viscoelastic material in Schlemm's canal when the microcathether encounters a stop during the cannulation; this may rupture the canal and cause descemet membrane detachment. This can lead to an intracorneal hematoma due to blood arising from the collector channels and Schlemm's canal and pooling in the descemet detachment, which may eventually require adjunctive surgical removal [17].

Small amounts of bleeding from Schlemm's canal in the anterior chamber is seen quite frequently within the first day of surgery, which could be a sign of positive prognosis considering that the blood arises from the collector channels and may be indicative that the outflow pathways are open and still functioning [18]. A rise in IOP can be seen in some cases during the early postoperative period. Very high transient IOP spikes seldom occur. This may be due the presence of residual amounts of hyaluronic acid in Schlemm's canal that does not permit adequate aqueous humor outflow across the trabeculodescemetic window and into the collector channels until it is completely washed away. IOP usually tends to stabilize within 24–48 hours when all traces of viscoelastic material are no longer present.

If the IOP remains high after three to four weeks, a YAG laser goniopuncture should be considered before the addition of topical medical treatment.

Long-term failure of canaloplasty is sometimes observed. In these cases, if a YAG laser goniopuncture fails to lower IOP and medical therapy is not sufficient or is poorly tolerated, a trabeculectomy can be a reasonable choice.

Considering the postoperative IOP values, the outcomes of canaloplasty appear to be superior to other well-established surgical techniques, such as viscocanalostomy [9], but statistically inferior to trabeculectomy with antimetabolites, in which postoperative IOP tends to lie in the lower teens [14]. Our results are quite similar to those reported by other authors. Our complete success rate after three years was very close to that of Lewis et al. [10], which, however, reported a higher qualified success rate (95.5% versus 86.2% for IOP ≤ 21 mmHg and 77.5% versus 58.8% for IOP ≤ 18 mmHg). Similar discrepancies can be found when comparing our results with those reported by Bull et al. [11].

The complication rate after canaloplasty tends to be much lower when compared with trabeculectomy, [14], especially considering the possible severe complications such as hypotonus with maculopathy (0% versus 4%) and choroidal effusion (0% versus 17%). In our cohort of patients, the only noteworthy complications observed included transient IOP spike >10 mmHg in nearly 6% of eyes, and Descemet membrane detachment (5.1% of cases), which resolved in a short period of time. In total, the complication rates in our cohort of patients were similar to those reported in previous canaloplasty studies [2, 3, 5, 6, 8, 10, 11]; only hypotonus was more frequently observed (9.8%) in comparison with other reports [10, 11]. One possible explanation could be due to sutures of the superficial scleral flap, which were nonperfectly watertight during the learning curve. However, this complication was transient and no case of maculopathy was observed.

In conclusion, canaloplasty is a demanding and rather difficult surgical technique, which provides very promising surgical outcomes. The technique is relatively new and literature in this field is limiting, thus improvements and future studies are surely needed to address the following issues: (1) specific criteria to determine which patients can benefit from this surgery; (2) instruments and tools to assess whether or not collector channels are still functioning [19–22]; and (3) simplification and standardization of the procedure. The postoperative results up to five years are very interesting. The rate of success is quite high and complications are seldom serious and sightthreatening. The main advantage of this blebless procedure is that physiological aqueous humor outflow is restored. Even eyes with chronic conjunctivitis arising from long-term antiglaucoma medical treatment or suffering from severe conjunctival scarring, differently from filtering techniques, can be considered for surgery. Another important advantage in comparison with trabeculectomy is that postoperative follow-ups tend to be simplified, less stringent, and fewer, which may render canaloplasty more cost-effective in the long run [23, 24].

In conclusion, canaloplasty appears to be a promising surgical procedure and can prove to be an important step towards a safer and more effective treatment in selected patients affected with various types of open-angle glaucoma. Further multicenter studies are needed, especially those reporting long-term results and complications.

## Figures and Tables

**Table 1:** Pre- and postoperative intraocular pressure measurements (mmHg).

	Preop	D1	W1	M1	M3	M6	M12	M18	M24	M30	M36	M42
No of eyes	214	214	214	214	207	194	144	129	80	69	29	19
Mean ± SD	29.4 ± 7.9	13.3 ± 6.1	17.3 ± 6.8	18.1 ± 7.4	17.1 ± 4.7	17.3 ± 4.8	16.8 ± 4.2	16.7 ± 4.0	17.1 ± 4.7	16.4 ± 4.7	17.3 ± 3.9	16.9 ± 3.1
95% CI	18.0–52.1	3.0–28.0	4.2–31.1	6.4–35.1	8.0–27.0	9.3–26.2	10.0–25.1	10.0–23.4	8.9–28.1	10.4–25.2	8.3–27.7	10.0–22.0
*P* value*		<0.0001	<0.0001	<0.0001	<0.0001	<0.0001	<0.0001	<0.0001	<0.0001	<0.0001	<0.0001	<0.0001

D: day; W: week; M: month: SD: standard deviation; CI: confindence interval; *paired *t*-test in comparison with the preoperative values.

**Table 2:** Success rate.

	≤21 mm/Hg		≤18 mm/Hg		≤16 mm/Hg	
	Qu	Co	Qu	Co	Qu	Co
	Number (%)	Number (%)	Number (%)	Number (%)	Number (%)	Number (%)
1 year (144 eyes)	128 (88.9)	75 (53.6)	108 (75.0)	64 (44.4)	68 (47.2)	49 (34.0)
2 years (80 eyes)	71 (88.7)	37 (46.2)	59 (73.7)	30 (37.5)	37 (46.2)	25 (31.2)
3 years (29 eyes)	25 (86.2)	13 (44.8)	17 (58.6)	9 (31.0)	11 (37.9)	7 (24.1)

Qu: qualified success; Co: complete success.

**Table 3:** Early complications after canaloplasty.

Complication	Number of cases (%)
(i) Hyphema	47 (21.9%)
(ii) Aqueous leakage from the conjuntival flap	2 (0.9%)
(iii) Hypotony <5 mmHg	21 (9.8%)
(iv) Transient IOP spike >10 mmHg	12 (5.6%)
(v) Descemet membrane detachment	11 (5.1%)
(vi) Suture cheese-wiring through trabecular meshwork	2 (0.9%)
(vii) Conjuntival bleb clinically detectable	3 (1.4%)
